# Steric Shielding of Surface Epitopes and Impaired Immune Recognition Induced by the Ebola Virus Glycoprotein

**DOI:** 10.1371/journal.ppat.1001098

**Published:** 2010-09-09

**Authors:** Joseph R. Francica, Angel Varela-Rohena, Andrew Medvec, Gabriela Plesa, James L. Riley, Paul Bates

**Affiliations:** 1 Department of Microbiology, University of Pennsylvania School of Medicine, Philadelphia, Pennsylvania, United States of America; 2 Department of Pathology and Lab Medicine and Abramson Family Cancer Research Institute, University of Pennsylvania School of Medicine, Philadelphia, Pennsylvania, United States of America; Mount Sinai School of Medicine, United States of America

## Abstract

Many viruses alter expression of proteins on the surface of infected cells including molecules important for immune recognition, such as the major histocompatibility complex (MHC) class I and II molecules. Virus-induced downregulation of surface proteins has been observed to occur by a variety of mechanisms including impaired transcription, blocks to synthesis, and increased turnover. Viral infection or transient expression of the Ebola virus (EBOV) glycoprotein (GP) was previously shown to result in loss of staining of various host cell surface proteins including MHC1 and β1 integrin; however, the mechanism responsible for this effect has not been delineated. In the present study we demonstrate that EBOV GP does not decrease surface levels of β1 integrin or MHC1, but rather impedes recognition by steric occlusion of these proteins on the cell surface. Furthermore, steric occlusion also occurs for epitopes on the EBOV glycoprotein itself. The occluded epitopes in host proteins and EBOV GP can be revealed by removal of the surface subunit of GP or by removal of surface N- and O- linked glycans, resulting in increased surface staining by flow cytometry. Importantly, expression of EBOV GP impairs CD8 T-cell recognition of MHC1 on antigen presenting cells. Glycan-mediated steric shielding of host cell surface proteins by EBOV GP represents a novel mechanism for a virus to affect host cell function, thereby escaping immune detection.

## Author Summary

The Ebola virus (EBOV) is a highly pathogenic virus that infects humans and non-human primates, causing severe disease or death in the majority of these cases. The interaction of this virus with its host on a cellular level is only just beginning to be understood. EBOV, like many viruses, affects the expression or function of several cell surface proteins, including adhesion factors and protein complexes responsible for allowing the immune system to recognize infected cells. Our group and others have previously shown that expression of the main viral glycoprotein of EBOV in cultured cells is sufficient to cause this disruption. Here we have identified the mechanism by which this disruption occurs. Heavily glycosylated domains of the EBOV glycoprotein form a steric shield over proteins at the cell surface. This steric interference blocks the detection of affected surface proteins using antibody reagents, but also has the functional effect of abrogating cell adhesion and preventing interactions with CD8 T cells. The results from this study highlight a novel mechanism for viral disruption of host cell surface protein functions and give insight to interactions between the Ebola virus and its host.

## Introduction

EBOV is an enveloped, negative-stranded RNA virus, a member of the family *Filoviridae*, and the causative agent of Ebola viral hemorrhagic fever. To date, five subtypes of EBOV have been identified: Zaire, Sudan, Côte d'Ivoire, Reston and Bundibugyo. EBOV Zaire is the most pathogenic subtype in humans, with mortality rates reaching 90% [1]. The basis for the high pathogenicity of EBOV is unclear, however immune dysregulation has been hypothesized to play a role [2]. Similarly to many other viral systems, EBOV infection appears to downmodulate the expression of host surface proteins involved in cellular recognition, most notably major histocompatibility complex (MHC) molecules and integrins [3].

EBOV encodes two forms of its glycoprotein. One is a dimeric, secreted form (sGP), which is transcribed directly from the viral RNA [4], [5] and whose function remains unclear. A second glycoprotein species results from transcriptional editing of the glycoprotein ORF and encodes a trimeric, membrane-bound form (GP). This form is expressed at the cell surface and is incorporated into the virion [4] and drives viral attachment and membrane fusion. GP is initially translated as a precursor (GP_0_), which is then cleaved by furin in the Golgi into two subunits, a surface subunit, GP_1_ and a membrane-spanning subunit, GP_2_ [6]. These subunits remain covalently connected through a single intermolecular cysteine bond [7]. Expression of the main viral glycoprotein, GP, has been shown to cause effects in cell culture on host surface proteins similar to those observed during viral infection, and so is proposed to be an important determinant of viral pathogenesis [8], [9], [10], [11]. Because sGP is the predominant form transcribed, it has been postulated that the balance between sGP and GP serves to regulate the cellular effects of GP [11].

Expression of high levels of EBOV GP in cultured cells disrupts cell adhesion resulting in loss of cell-cell contacts as well as cell rounding and loss of attachment to the culture substrate [8], [10], [12]. This can be observed in a variety of cell lines and primary cell types [12]. Interestingly, while transient GP expression does not cause death in human embryonic kidney 293T cells, primary human cardiac microvascular endothelial cells have been reported to undergo anoikis, or detachment-mediated apoptosis, upon transduction of GP [12], [13]. By flow cytometry, cells expressing GP display dramatically lowered levels of various surface proteins, including several members of the integrin family and MHC class I (MHC1); however, the exact complement of surface proteins affected by GP appears to differ by cell type [10], [12], [14]. Importantly, EBOV infection of 293T cells was observed to cause similar reduction of β1 integrin and MHC1 staining by flow cytometry, confirming that observations from transient GP expression are not simply artifacts of overexpression [3]. The effects of EBOV GP are caused by a highly glycosylated region in GP_1_, the mucin domain [8], [12], [14]. This domain encompasses approximately 150 amino acids, contains numerous N- and O- linked glycosylation sites, and is a distinctive feature of filoviral GPs. The mucin domain is not only necessary, but also sufficient for the observed EBOV GP-mediated effects upon surface protein expression and cellular adhesion [8], [15].

Few studies have been undertaken to investigate the mechanism by which EBOV GP disrupts adhesion and causes surface protein downmodulation. Our recent analysis concluded that the cellular endocytic factor dynamin does not play a role in surface protein downmodulation, suggesting the process may not involve cycling of proteins from the cell surface [15]. In contrast, Sullivan and colleagues have reported that this process requires dynamin [14]. Additionally, it has been reported that the extracellular signal-regulated kinases (ERK 1/2) play a role in downmodulation [16] suggesting an active process. In the present study, we provide direct evidence that EBOV GP-mediated loss of surface protein recognition occurs via steric shielding of surface epitopes, not by protein removal from the cell surface. Moreover, we demonstrate that EBOV GP expression blocks MHC1-mediated stimulation of T cells. Based upon these findings, we present a model in which the heavily glycosylated EBOV glycoprotein acts as a “glycan umbrella” to physically occlude access to host proteins, and GP itself, thereby impairing host protein function. EBOV GP-mediated steric occlusion represents a unique viral mechanism to interfere with the function of host proteins.

## Results

### EBOV GP expression blocks surface protein staining

**Figure 1:**
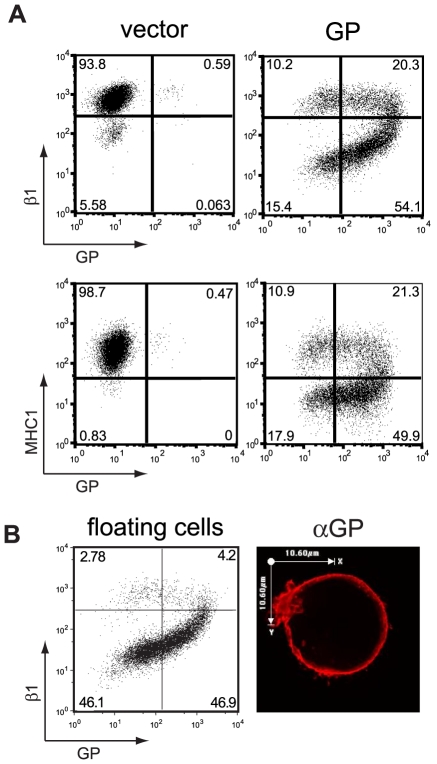
Transient expression of the EBOV glycoprotein results in loss of surface staining of β1 integrin and MHC1. (A) 293T cells were transfected with empty pCAGGS (vector) or vector encoding GP. Floating and adherent cells were harvested 24 h after transfection, pooled, and stained for GP using the KZ52 antibody, followed by FITC-labeled secondary antibodies, and co-stained for β1 integrin or MHC1 with PE-Cy5 conjugated monoclonal antibodies and assayed by flow cytometry. (B) Following transfection with vector encoding GP, floating 293T cells were removed from adherent cells, stained for β1 integrin and assayed by flow cytometry (left panel). Similarly treated cells were mounted on coverslips, fixed, permeabilized and stained for GP with mouse monoclonal antibodies, followed by Alexa 594 conjugated antibodies and assayed by immunofluorescence microscopy. A representative cell is shown (right panel).

EBOV GP expression can dramatically reduce the observed levels of numerous host cell surface proteins including factors involved in immune recognition and cellular adhesion [10], [12], [14]. This effect can be seen by analysis of MHC1 or β1 integrin by flow cytometry staining in HEK293T cells transiently expressing Zaire EBOV GP (Figure 1A). Overall, a 10- to 50-fold reduction in surface levels of these host markers is observed in cells transfected with an EBOV GP cDNA. Additionally, there appears to be a critical threshold of EBOV GP expression required to induce surface protein downmodulation [15]. In parallel with the decrease in staining for host proteins, EBOV GP expression also appears to be reduced, resulting in a distinctive horseshoe-shaped flow cytometry profile (Figure 1A and [14], [15], [16]). Despite this apparent decrease in surface protein levels observed by flow cytometry, there were no consistent, significant changes in total protein levels for the EBOV glycoprotein upon analysis by Western blot in either adherent or non-adherent EBOV GP transfected cells (data not shown). To look directly at host protein expression in cells expressing EBOV GP, nonadherent, GP-transfected 293T cells were collected and analyzed by flow cytometry for expression of β1 integrin ([15] and Figure 1B, left panel). As previously described [15], these nonadherent cells represent the lower two quadrants of the “horseshoe” and appear to have reduced levels of both β1 integrin and EBOV GP. In contrast to the flow cytometry results, analysis of EBOV GP in these cells by immunofluorescence microscopy after fixation and permeabilization reveals extensive staining at the plasma membrane (Figure 1B, right panel). Similar to these results, previously published microscopic analysis of cells expressing EBOV GP also shows extensive plasma membrane staining with little evidence of significant accumulation of GP in internal vesicles [15], [17].

**Figure 2:**
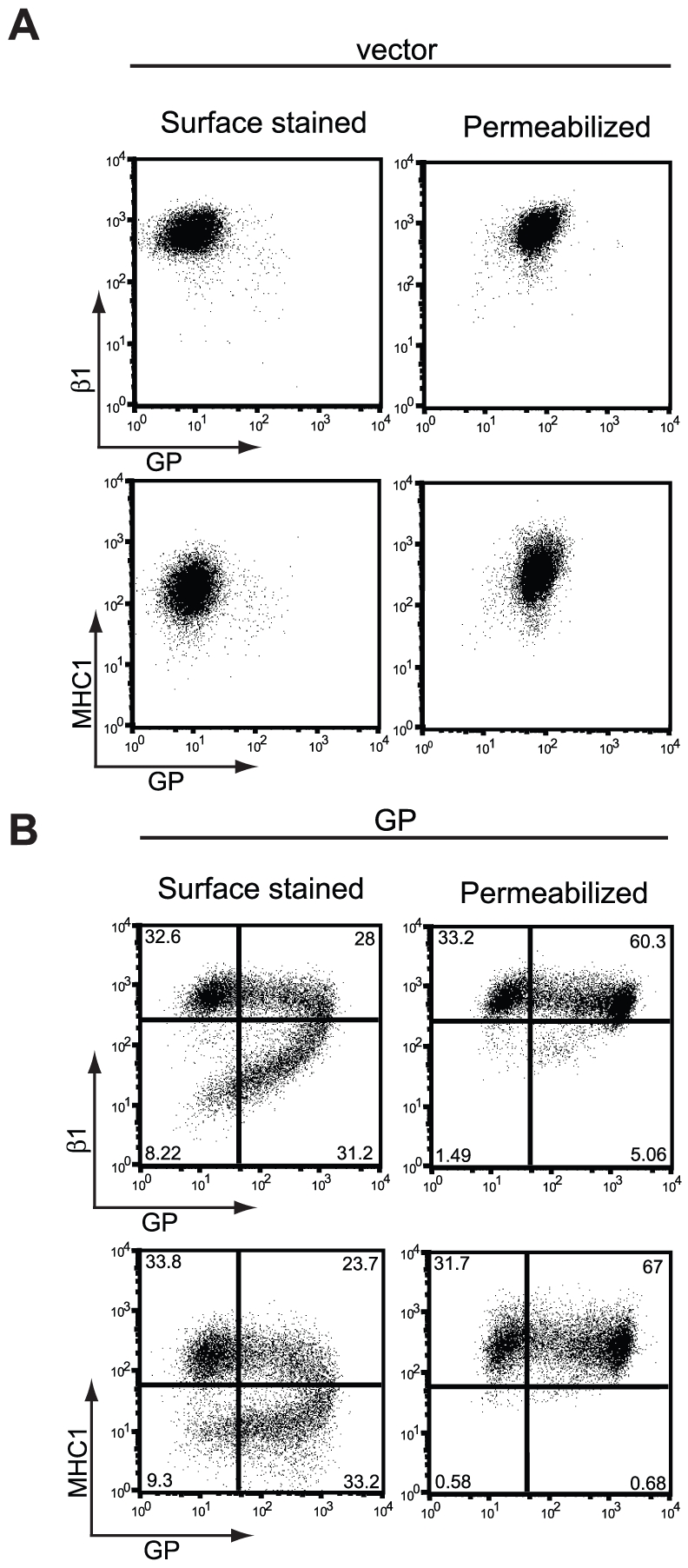
Steady-state levels of β1 integrin and MHC1 are unchanged in GP-expressing cells. 293T cells were transfected with empty vector (A) or vector encoding wt GP (B). Floating and adherent cells were harvested 24 h after transfection, pooled, and stained for GP, β1 integrin, and MHC1 as described earlier and assayed by flow cytometry. Prior to staining, a portion of cells were fixed and permeabilized to expose occluded surface and internal epitopes.

To evaluate steady state levels of host proteins and EBOV GP in cells transiently expressing the viral glycoprotein, the transfected cells were fixed, permeabilized and analyzed by flow cytometry. In vector-transfected cells, the permeabilization treatment had little effect upon staining for β1 integrin or MHC1 (Figure 2A). However, in cells transiently expressing EBOV GP, which displayed dramatically reduced levels of β1 integrin and MHC1 by surface staining (Figure 2B, left column), fixation and permeabilization reveals no decrease in either of these host proteins (Figure 2B, right column). Similarly, the apparent loss of EBOV GP staining is reversed by this treatment. These effects are best illustrated by comparison of the lower two panels in Figure 2B where without treatment, 9.3% of the cells displayed low MHC1 and EBOV GP levels, however after fixation and permeabilization the number of double negative cells was reduced to background levels and these now appear as MHC^+^, GP^+^ cells in the upper right quadrant. As expected, the untransfected cell population of 32–34% remains unaltered by this treatment (Figure 2B, upper left quadrants). Overall, this analysis suggests that the apparent downmodulation observed is not due to reduced steady-state levels of protein. Rather these transfected cells express unaltered levels of EBOV GP and MHC1, however these proteins are inaccessible for surface staining.

### EBOV GP shields its own epitopes at the cell surface

Recent structural analysis of EBOV GP suggests that the recognition site for the monoclonal antibody, KZ52, employed in the flow cytometry analysis resides near the base of the protein [18] below the globular GP_1_ and heavily glycosylated mucin domains in GP. This finding, coupled with our results suggesting that downmodulation in these cells was not accompanied by a reduction in steady-state levels of β1 integrin or MHC1, or a significant re-localization of EBOV GP, prompted us to consider the hypothesis that EBOV GP mediates its effects by blocking access to epitopes of proteins on the cell surface including epitopes within GP. Additionally, this hypothesis is consistent with the apparent threshold of GP expression required for downmodulation as well as the lack of a dynamin requirement [15].

**Figure 3:**
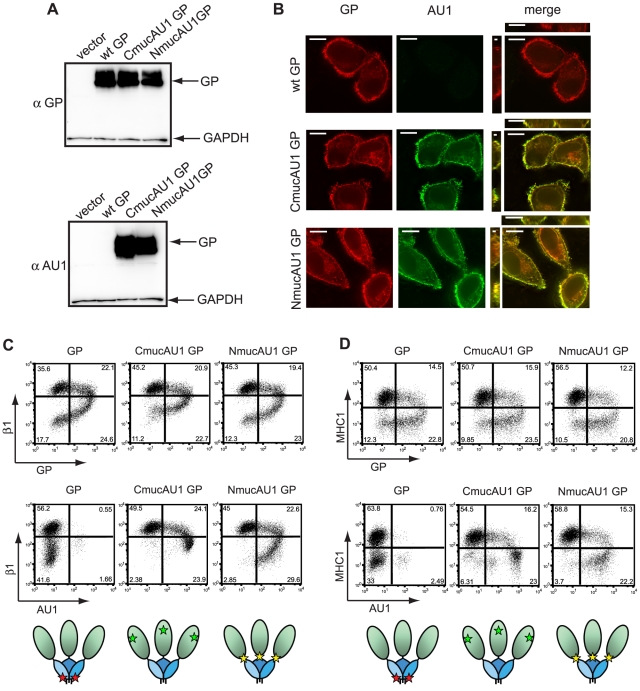
The mucin and globular domains of the EBOV glycoprotein masks the KZ52 epitope on the cell surface. (A) 293T cells were transfected with empty vector or vector encoding wt GP, CmucAU1 GP, or NmucAU1 GP. Lysates were harvested in RIPA buffer after 24 h and subjected to SDS-4 to 15% PAGE, transferred to PVDF, and immunoblotted with anti-GP polyclonal rabbit antibodies (top blot) or anti-AU1 antibodies (bottom blot) and anti-GAPDH antibodies. (B) HeLa cells were transfected with vector encoding wt GP, CmucAU1 GP, or NmucAU1 GP. 24 h after transfection, cells were fixed, permeabilized, and stained for GP with mouse monoclonal antibodies, and the AU1 epitope with anti-AU1 antibodies, followed by Alexa 594 and Alexa 488 conjugated antibodies, respectively and assayed by immunofluorescence microscopy. Scale bars are 10.6 µm. (C, D) 293T cells were transfected with vector encoding wt GP, CmucAU1 GP, or NmucAU1 GP. Floating and adherent cells were harvested 24 h after transfection, pooled, and stained for GP using the KZ52 antibody or the AU1 tag, β1 integrin, and MHC1, as described earlier and assayed by flow cytometry. (C) β1 integrin vs. GP or AU1 surface staining. (D) MHC1 vs. GP or AU1 surface staining. Cartoon depictions of the KZ52 epitope (red star), CmucAU1 epitope (green star) or NmucAU1 epitope (yellow star) are shown below their respective flow cytometry plots. The globular region of GP is shown shaded blue; the mucin domain is shown shaded green.

To test this hypothesis, we engineered epitopes within EBOV GP at locations which, based on their position relative to the mucin domain and the globular region of GP, are predicted to be more accessible than the KZ52 epitope. Two constructs were created with an AU1 antibody epitope tag at the N or C terminus of the mucin-like domain, termed NmucAU1 GP and CmucAU1 GP, respectively. Cartoon depictions of each construct are shown in Figure 3C and D. These constructs were well expressed, as judged by Western blot analysis for EBOV GP and the AU1 tag (Figure 3A). The sub-cellular localization of these constructs was also evaluated in HeLa cells by immunofluorescence microscopy and was found to be indistinguishable from wt GP (Figure 3B).

Although the structure of the mucin domain is unknown, its mucin-like O glycosylation may force the domain into an extended conformation as has been suggested for cellular mucin proteins [19]. This would likely position the C terminus of the mucin domain so that it is more exposed compared to the GP core (Figure 3C and D). Based upon the proposed steric occlusion model, we hypothesized that the AU1 epitope of CmucAU1 would be most accessible to antibody staining. In contrast, the AU1 epitope in NmucAU1 might be less accessible than the epitope in CmucAU1 because of its location at the base of the mucin domain. Cells expressing wt GP, CmucAU1 GP, and NmucAU1 GP were analyzed by flow cytometry. When stained with the GP-specific KZ52 antibody, the epitope-tagged mutants displayed the characteristic comma-shaped flow cytometry plot seen with wt GP (Figure 3C and D; top rows). In contrast to the reduced KZ52 staining observed, the AU1 epitope in CmucAU1 was highly visible by flow cytometry (Figure 3C and D; bottom middle panels). Staining of the AU1 epitope on NmucAU1 GP was intermediate relative to CmucAU1 GP and wt GP KZ52 staining (Figure 3C and D; bottom right panels). In support of the shielding model, these data demonstrate that cells exhibiting reduced levels of β1 integrin and MHC1 have high surface levels of GP as revealed by AU1 staining, not reduced levels as indicated by KZ52 staining. Furthermore, these data suggest that antibody accessibility to epitopes in GP differs based on the epitope position relative to the mucin domain and the globular regions of GP_1_.

### Removal of the EBOV GP_1_ subunit reveals shielded host surface proteins

**Figure 4:**
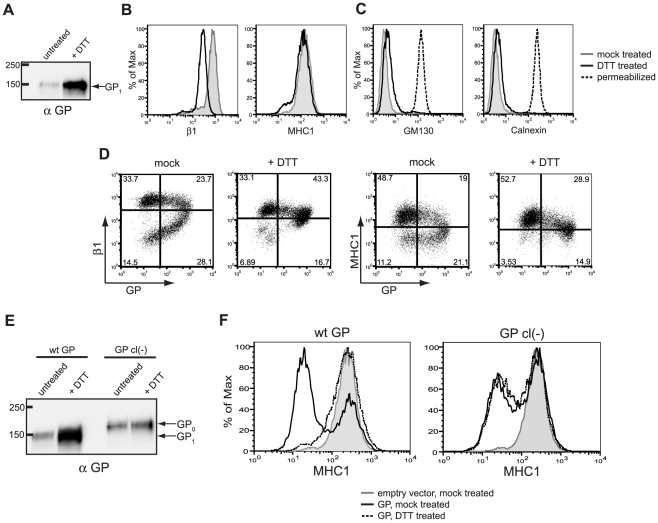
Removal of the GP_1_ subunit from the cell surface results in exposure of previously occluded surface epitopes. (A-C) 293T cells were transfected with empty vector or vector encoding wt GP. Floating and adherent cells were harvested 24 h after transfection, pooled, and either left untreated, or incubated at 37°C for 20 minutes in 150 mM DTT. (A) Western blot analysis of the GP_1_ subunit shed into the supernatant of untreated or DTT-treated cells. (B) Cells were transfected with empty vector and assayed by flow cytometry to show baseline differences in surface staining for β1 integrin and MHC1 between untreated cells (grey shading) and DTT-treated cells (black trace). (C) Cells were transfected with vector encoding wt GP and assayed by flow cytometry for the internal proteins GM130 and calnexin to show the effect of DTT on cell permeabilization. Untreated cells are shown in the grey shading; DTT-treated cells are shown in the black trace, and, as a positive control, fixed/permeabilized cells are shown in the dashed trace. (D) Cells were transfected with vector encoding wt GP, were mock- or DTT-treated, stained for GP and β1 integrin or MHC1 as described above, and assayed by flow cytometry. (E, F) 293T cells were transfected with empty vector or vector encoding wt GP or GP cl(-). Cells were harvested and treated as above. (E) Western blot analysis using rabbit polyclonal antibodies of GP_1_ or GP_0_ shed into the supernatant of untreated or DTT-treated cells. (F) Transfected and treated cells were surface stained for MHC1 and assayed by flow cytometry. Empty vector-transfected, untreated cells are shown in the grey shading; GP-expressing cells are shown after mock treatment (black trace) or DTT treatment (dashed trace).

The data presented above are consistent with EBOV GP affecting recognition of epitopes within GP by shielding, however we wished to address if a similar mechanism was responsible for the apparent downmodulation of host surface proteins. To directly address whether EBOV GP sterically occludes host surface protein epitopes, we sought to unmask MHC1 and β1 integrin staining. We hypothesized that dissociation of the GP_1_ subunit, which includes the mucin domain and globular “head” region of EBOV GP, from GP_2_ at the cell surface should relieve the shielding of previously occluded epitopes. The GP_1_ subunit is covalently linked to GP_2_ via a single sulfahydryl bridge between residues C53 and C609 [7]. We have previously demonstrated that this bond can be reduced by incubation with DTT, allowing for dissociation of the EBOV GP_1_ subunit from the surface of virions [20]. To confirm that DTT is able to effectively remove GP_1_ from the cell surface, cells expressing GP were incubated with DTT then the supernatant was analyzed for GP by Western blot. Figure 4A reveals that GP_1_ was readily detected in the supernatant of cells incubated with DTT compared to mock treated cells. Control experiments also demonstrated that the DTT treatment did not significantly alter surface expression of β1 integrin or MHC1 in mock-transfected cells (Figure 4B). Moreover, this treatment did not result in permeabilization of the cells (Figure 4C) which, as shown above (Figure 2), could also rescue β1 integrin and MHC1 staining. In addition, these and the following experiments were carried out in the presence of azide and 2-deoxy glucose to ensure that the trafficking of nascent or recycled protein did not complicate the interpretation of this assay.

We next examined the effect of DTT treatment on surface staining of β1 integrin and MHC1 in cells expressing EBOV GP. Flow cytometry analysis of the DTT-treated, GP-expressing cells indicates that GP-induced loss of staining of β1 integrin and MHC1 is reversed by DTT treatment and subsequent dissociation of GP_1_ from the cells: upon DTT treatment, staining of β1 integrin and MHC1 is restored to nearly control levels (Figure 4D). Interestingly, staining for GP was also rescued, resulting in cells that stained positively for both GP and β1 integrin or MHC1. This is somewhat counter-intuitive, as KZ52 makes critical contacts with residues on GP_1_ [18], which is removed from the cell surface by DTT. These data suggest that DTT treatment removes a significant amount of GP_1_ from the cell surface – enough to reverse the steric occlusion of β1 integrin and MHC1 epitopes, as well as the KZ52 epitope. However, sufficient GP_1_ remains on the cell surface to allow for staining of GP by flow cytometry. Supporting this hypothesis, there appears to be a very modest downmodulation of integrin and MHC1 on the cells that appear to have the highest levels of GP (Figure 4D, upper right quadrant of the +DTT flow cytometry plots). This finding agrees with our previously published study that suggests a threshold level of EBOV GP is needed to downmodulate β1 integrin, MHC1 or GP [15].

Removal of surface GP_1_ by DTT reverses the apparent downmodulation of surface proteins induced by EBOV GP. To ensure this effect could be directly attributed to the EBOV glycoprotein we tested the effect of DTT on cells expressing a mutant form of GP lacking the endoproteolytic site required for processing GP_0_ into GP_1_ and GP_2_ subunits. Previous analysis demonstrated that this mutant EBOV glycoprotein, GP cl(-), retains normal viral entry function [20], [21] and is therefore likely folded similarly to wt EBOV GP. As shown in Figure 4F, GP cl(-) also downmodulates MHC1 similarly to wt EBOV GP. However in contrast to wt GP, DTT treatment of cells expressing this uncleaved form of GP does not relieve the observed downmodulation of MHC1 (Figure 4F). As anticipated, DTT treatment of cells expressing GP cl(-) produced no increase in GP release compared to untreated cells (Figure 4E). The EBOV glycoprotein found in the supernatant from the GP cl(-) expressing cells likely represents trimeric GP released by the cellular enzyme TACE [22]. Overall, these data strongly support the model proposed for EBOV GP mediated occlusion of host surface proteins.

### Carbohydrate modification of GP is important for steric shielding

**Figure 5:**
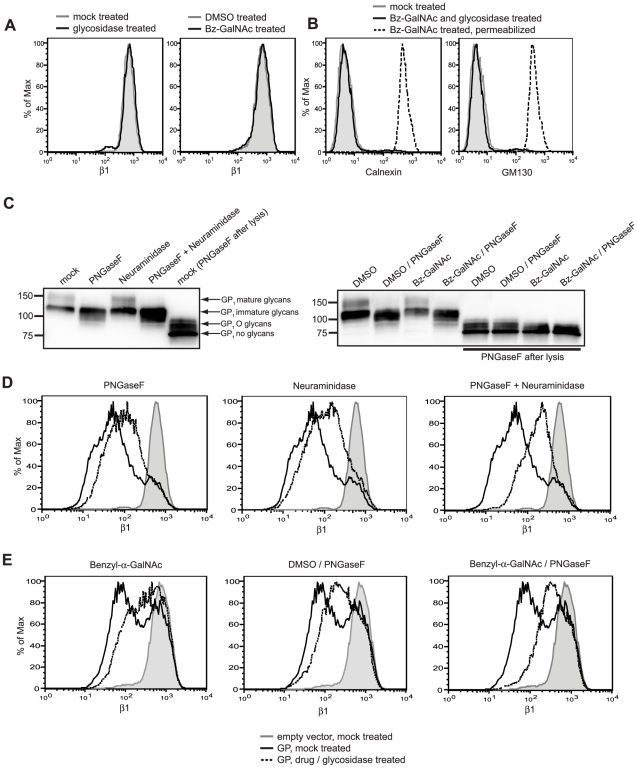
Surface N- and O- linked glycans contribute to GP-mediated shielding. (A, left plot) Cells transfected with empty vector were mock incubated (grey shading) or incubated with neuraminidase and PNGaseF (black trace), stained for β1 integrin and assayed by flow cytometry. (A, right plot) Cells were treated with DMSO, transfected with empty vector, and mock incubated (grey shading) or treated with benzyl-α-GalNAc (bz-GalNAc), transfected with empty vector, and incubated with PNGaseF (black trace), then stained for β1 integrin and assayed by flow cytometry. (B) Cells were left untreated and untransfected (grey shading), or treated with bzGalNAc, transfected with vector encoding GP, and incubated with PNGaseF (black trace), then assayed by flow cytometry for the internal proteins GM130 and calnexin to show the effect of these treatments on cell permeabilization. As a positive control, bz-GalNAc-treated, fixed/permeabilized cells are shown (dashed trace). (C, D) Floating cells in cultures transfected with vector encoding GP were mock incubated or incubated with neuraminidase and/or PNGaseF, then analyzed by Western blot with rabbit polyclonal antibodies to GP (C, left blot) or stained for β1 integrin and assayed by flow cytometry (D). (C, E) Cells were treated with DMSO or bz-GalNAc, then transfected with empty vector, or vector encoding GP. Cells were then mock-incubated, or incubated with PNGaseF, then analyzed by Western blot (C, right blot) or stained for β1 integrin and assayed by flow cytometry (E). For D and E, mock-incubated cells or DMSO-treated cells transfected with empty vector =  grey shading; GP-transfected and DMSO-treated or mock-incubated cells =  black traces; GP-transfected and bz-GalNAc- and glycosidase-treated cells =  dashed traces. In Western blot panels (C), selected samples were lysed and denatured before incubation with PNGaseF for comparison, as indicated.

EBOV GP is a heavily glycosylated protein, and we have previously shown the mucin domain to be sufficient to induce loss of staining of host surface proteins by flow cytometry [15]. Therefore, we directly addressed whether GP glycosylation plays a role in the shielding of surface epitopes. GP-expressing cells were treated with several glycosidases or pre-treated with a small molecule inhibitor of mucin synthesis, benzyl-α-GalNAc, then assayed for β1 integrin staining by flow cytometry. Importantly, none of the glycan-interfering treatments used here increased the staining for β1 integrin in cells transfected with empty vector (Figure 5A). Also, these treatments did not cause the permeabilization of cells, allowing us to attribute changes in staining to alterations at the cell surface (Figure 5B). Staining for β1 integrin on GP-expressing cells was increased by incubation with PNGaseF, an endoglycosidase that cleaves N-linked sugar moieties (Figure 5D left). Similarly, staining for β1 integrin was increased by incubation with neuraminidase, an exoglycosidase that cleaves sialic acid, which is a common component of mucin sugars. (Figure 5D, middle). When GP-expressing cells were incubated with both PNGaseF and neuraminidase, an additive effect was seen and β1 integrin staining was further increased (Figure 5D, right). The effect of glycosidase treatment on cellular GP was also analyzed by Western blot (Figure 5C, left). PNGaseF treatment results in loss of the top band of GP_1_, which is the maturely-glycosylated form and the appearance of bands which co-migrate with GP_1_ that has been PNGaseF treated under denaturing conditions, but which still contains O glycosylation. Treatment with neuraminidase did not result in a perceivable shift in migration of GP_1_; this is likely due to the small mass of these glycans and the resolution of the gel. These data indicate a direct role for N-linked glycans in GP-mediated loss of β1 integrin staining.

To directly address the role of O glycosylation in host protein downmodulation by EBOV GP, O glycosylation was perturbed by pre-incubating cells with benzyl-α-GalNAc or the control vehicle DMSO. This compound is a competitive inhibitor of β1,3-galactosyltransferase, which prevents the modification of core O glycan structures, resulting in shorter O-linked glycans and reduced sialyation [23], [24], [25]. Cells pre-treated with benzyl-α-GalNAc, then transfected with vector encoding GP showed increased staining for β1 integrin compared to DMSO treated cells, consistent with a role for O glycoslyation in the shielding of epitopes by the GP mucin domain (Figure 5E, left plot). In cells pre-treated with benzyl-α-GalNAc and expressing GP, incubation with PNGaseF further increased staining for β1 integrin (Figure 5E, right plot). Cells pre-treated with benzyl-α-GalNAc were also incubated with O-glycosidase, which can cleave unmodified core GalNAc structures; however, no further increase in β1 integrin was observed (data not shown). This is perhaps due to remaining modification of the core O glycans. The effect of these treatments on GP glycosylation was analyzed by Western blot (Figure 5C, right blot). Treatment with benzyl-α-GalNAc results in a modest increase in mobility for bands corresponding to GP containing O glycosylation, which are most easily seen in samples that have been PNGase-treated after cell lysis. Our data here suggest that the mass of O glycosylation is reduced, but not fully eliminated. This is expected, as benzyl-α-GalNAc only reduces mucin modification, but does not prevent the synthesis of initial core glycans. Taken together, these data demonstrate that surface N- and O- linked glycans, presumably on EBOV GP, contribute to the ability of GP to mask surface β1 integrin epitopes.

### EBOV GP expression blocks MHC1 mediated T-cell activation

**Figure 6:**
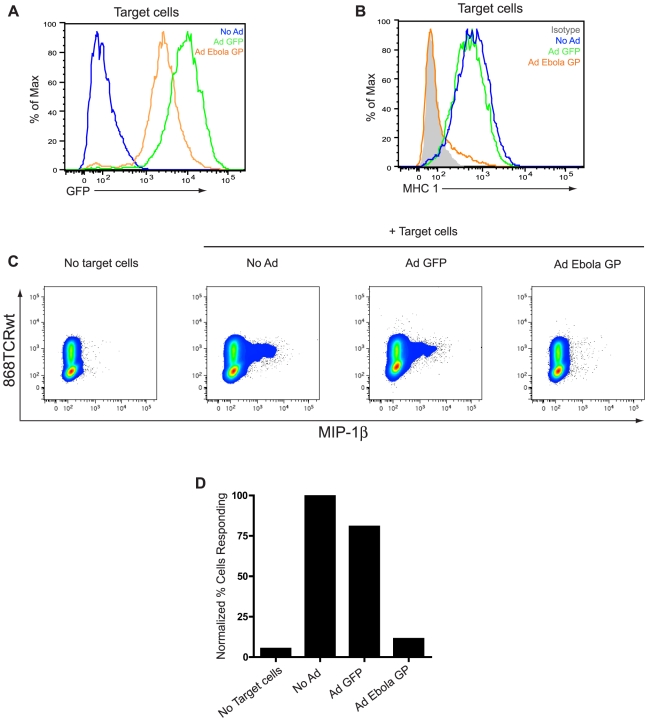
EBOV GP-induced disruption of MHC1 prevents activation of CD8^+^ T cells. OV79 SL9 target cells were mock transduced (no Ad) or transduced with Adenoviral vectors expressing GFP (Ad GFP) or GFP and EBOV GP (Ad GP) at an MOI of 300. 48 h after transduction, cells were assayed for GFP expression (A); No Ad =  blue trace; Ad GFP =  green trace; Ad GP =  orange trace. Cells were also stained for MHC1 (B); isotype antibody =  shaded peak; No Ad =  blue trace; Ad GFP =  green trace; Ad GP =  orange trace. In parallel, CD8 T cells expressing a transgenic TCR (868TCRwt) that recognizes the SL9 HLA-A2 complex were incubated alone or with mock- (no Ad) or Ad- transduced target cells in a 2∶1 ratio. After co-culture, T cells were surface stained for CD8, then fixed and permeabilized, and stained for 868TCRwt and MIP-1β with APC-H7, FITC, and PE- conjugated antibodies, respectively, and assayed by flow cytometry. (C) CD8^+^ and 868TCRwt^+^ events were analyzed for MIP-1β staining. (D) Bar graph depicts percent cells positive for MIP-1β, normalized to the No Ad target cell sample.

The data presented above demonstrate that epitopes at different locations within EBOV GP are differentially occluded in GP expressing cells (Figure 3). To determine if a similar situation occurs for cellular proteins, four monoclonal antibodies that recognize distinct regions of MHC1 were analyzed. In cells expressing GP, staining for MHC1 is blocked regardless of the epitope examined (Supplemental Figure S1). Given the ability of EBOV GP to effectively mask all of the epitopes examined on MHC1, we wanted to address whether this had functional consequences for MHC1. Human OV79 cells expressing the HIV Gag-derived peptide SLYNTVATL (SL9) were used to test the effect of EBOV GP on MHC1 antigen presentation. These cells present the SL9 antigen using a stably expressed MHC1, HLA-A2. The OV79- SL9 cells were mock transduced or transduced with adenoviral vectors encoding GFP (AdGFP) or GFP and EBOV GP (AdGP), which resulted in nearly 100% of cells expressing GFP (Figure 6A). Expression of EBOV GP dramatically reduced MHC1 levels in these cells whereas the control AdGFP vector had no effect on MHC1 expression (Figure 6B). Primary human CD8 T-cells transduced with a lentiviral vector expressing a T-cell receptor (868TCRwt) specific for SL9 were used to assess antigen presentation by GP-expressing OV79 cells. T-cell activation was measured by intracellular staining for production of the cytokine MIP-1β in CD8^+^ 868TCRwt^+^ expressing cells (Figure 6C). Production of MIP-1β has been shown to be the most sensitive indicator of HIV-specific CD8 T-cell activation [26]. Quantification of the CD8 activation results demonstrates that expression of EBOV GP had a profound effect on antigen presentation by the target cells, reducing T-cell responses to nearly background levels (Figure 6D). In contrast, the AdGFP control cells only modestly reduced the number of responding T-cells. Similar results were obtained using 293T target cells (data not shown). The ability of EBOV GP to interfere with T-cell function does not appear to be caused by inhibitory signaling from the GP-expressing antigen presenting cells (APCs) since T-cells were fully activated when anti-CD3/CD28-coated beads were added to a mixture of GP-expressing OV79 and CD8^+^ 868TCRwt^+^ cells (data not shown). Given the observations that all of the queried epitopes on MHC1 were occluded by GP expression and that T-cells are not stimulated by these APCs, the most straightforward explanation is that EBOV GP expression impedes T-cell recognition of antigen presenting cells. Overall these data support a model in which EBOV GP not only masks MHC and other surface proteins from antibody recognition, it also functionally inactivates them.

## Discussion

An important component of the virus host interaction is viral modulation of host functions. Many viruses alter expression and/or function of host surface proteins to affect signaling, immune surveillance, or viral superinfection. EBOV GP expression in cell culture has been observed by several groups to cause dramatic changes in cell adhesion and reduction in surface protein staining by flow cytometry [10], [12], [14], [16]. EBOV infection causes a similar reduction of β1 integrin and MHC1 staining by flow cytometry, suggesting that observations from transient GP expression are not simply artifacts of overexpression [3]. EBOV GP-induced effects have previously been assumed to result from removal of surface proteins from the plasma membrane. In this study we analyzed the mechanism of downmodulation of host surface proteins by the Ebola viral glycoprotein, GP. We show that reduction in surface staining for the host proteins MHC1 and β1 integrin is not accompanied by decreases in the total cellular levels of these proteins. Moreover, the observed self downmodulation of EBOV GP does not result in relocalization of GP away from the plasma membrane. Using epitopes placed at various locations in EBOV GP we find that the observed GP surface levels appear to differ based on epitope position relative to the mucin domain and the globular regions of EBOV GP. A similar observation has been made using a series of monoclonal antibodies to EBOV GP [27]. Additionally, the apparent downmodulation of surface proteins is reversed by removal of the EBOV GP_1_ subunit by reduction or by enzymatic digestion of the carbohydrate modification on EBOV GP. Finally, our data demonstrate that EBOV GP expression dramatically impairs antigen presentation by host cells. Taken together these data support a model in which EBOV GP utilizes a steric occlusion mechanism to downmodulate accessibility and function of host surface proteins.

The ability of viruses to affect host surface proteins has been well documented. For example, viruses may downregulate their cellular receptor, as in the case of HIV downregulation of CD4 and measles virus downregulation of the complement regulatory protein [28], [29]. Other common targets for virus mediated downmodulation are surface proteins related to immune surveillance. MHC1 is known to be downregulated from the cell surface by many viral proteins: HIV nef, Adenovirus E19, and KSHV K3 and K5, to name a few [30], [31], [32]. Activating ligands for natural killer (NK) cells have also been shown to be actively downregulated by KSHV and Hepatitis C virus [33], [34]. Multiple mechanisms and cellular pathways have been implicated in viral dysregulation of the various host surface molecules (reviewed for MHC1 in [35]). The model demonstrated here of glycan mediated steric occlusion by EBOV GP represents, to our knowledge, a distinctive mechanism for viral regulation of host surface proteins. Indeed, a similar steric masking model has recently been proposed for EBOV GP [27]. The polydnavirus, *Microplitis demolitor* bracovirus expresses a mucin domain-containing glycoprotein which can abrogate cell adhesion and thus may utilize a mechanism similar to that proposed here for EBOV [36].

Our observation that enzymatic removal of carbohydrate modification can relieve downmodulation, coupled with prior observations that the mucin domain of EBOV GP is sufficient for downregulation [8], [15], suggests that the steric occlusion observed is mediated, at least in part, by N- and O-linked modification of EBOV GP. A similar glycan mediated steric hindrance model has been proposed for cellular mucin proteins, which can disrupt a variety of cell-cell interactions at the plasma membrane [37], [38], [39], [40], [41]. For the cellular mucin proteins, densely-arrayed O-linked glycans are critical for disruption of cell adhesion, with different core glycan structure and subsequent modifications influencing the function and anti-adhesive properties of the protein [42]. Additionally, the number of mucin tandem repeats positively correlates with the anti-adhesive properties of Muc1 [41]. Similarly, we have shown that sequential removal of glycosylation sites in the mucin domain of EBOV GP led to a step-wise reduction in cell detachment suggesting that such modifications within GP are involved in downmodulation [12]. The O-linked glycosylation found on the EBOV GP mucin domain may promote an extended conformation as is seen for cellular mucin proteins [19] allowing this domain in GP to act as an approximately 150 residue long flexible rod that can protrude and mask epitopes in the immediate vicinity.

The ability of carbohydrate modification to protect epitopes on the surface of a viral glycoprotein is well established. Indeed, a glycan shield model has been proposed for other viral glycoproteins, most notably HIV, as a mechanism to avoid host immune recognition [43]. An extended glycosylated protrusion provided by the mucin domain may be a characteristic feature that distinguishes the “glycan umbrella” of EBOV GP from other viral glycoproteins where the glycan shield does not cause steric occlusion of host factors. Another feature of the proposed model is that EBOV GP must localize in close proximity to the affected proteins; perhaps within plasma membrane microdomains inhabited by the host proteins. This requirement may explain the critical threshold for the observed GP effects as well as the variety of proteins regulated by EBOV GP. It may be that the ability to occupy these microdomains is, in addition to the extensive carbohydrate modification, a characteristic feature of EBOV GP. Based upon our results it appears likely, therefore, that the heavily glycosylated EBOV GP acts as a glycan umbrella to physically occlude access to nearby host proteins, and GP itself, thereby impairing host protein function.

It is intriguing to consider the role in EBOV replication or pathogenesis of GP-induced steric occlusion of surface proteins. Based upon our observations of proteins at the plasma membrane it is plausible that EBOV GP functions to shield epitopes on the surface of virions thereby contributing to infection and/or persistence in the natural reservoir. Notably the KZ52 monoclonal antibody employed in these studies is neutralizing but fails to protect nonhuman primates from EBOV infection [44], [45]. Perhaps variation in GP density on virions produced in vivo differentially affects the neutralization sensitivity of viruses in nonhuman primates. Additionally, the ability of GP to mask MHC1 and other molecules on the cell surface, coupled with the inhibitory effect of GP on cell-cell adhesion, may be a strategy for avoiding CD8 T cell-mediated killing of EBOV infected cells. Our data demonstrating that GP-expressing cells do not effectively activate CD8 T cells supports this hypothesis. Interestingly, this mechanism is proposed for adenocarcinomas, in which cellular mucin protein overexpression can result in metastasis due to loss of adhesion, and has been shown to prevent recognition and killing by NK and cytotoxic T cells [38], [46], [47]. However, the rapid time course of EBOV infection and its impairment of adaptive responses may render escape from CD8 cells unnecessary in humans. Instead, protection from NK cells may be more important and the ability of EBOV GP to effect NK cell recognition should be explored. Alternatively, the ability to mask MHC1 may be more critical for viral infection or persistence in the natural reservoir for EBOV. Finally, it is known that the interface between the innate and adaptive immune response is affected during EBOV infection (reviewed in [2]). We have previously shown that EBOV GP causes rounding in macrophages [12]. It is possible that EBOV GP shielding and inhibition of adhesion molecules or other immune regulatory proteins on professional antigen presenting cells such as macrophage or dendritic cells plays a role in the immune dysfunction characteristic of EBOV infection.

## Materials and Methods

### Plasmids and transfections

For GP studies, cDNA encoding the membrane-anchored form of Zaire EBOV GP (Mayinga strain, accession number U23187) was used. For AU1 tagged GPs, the amino acids, DTYRYI were added using linker insertion into GP that had been engineered to have a unique XhoI site at position 312 encoding the amino acids LE (NmucAU1) and a unique NotI site replacing amino acid 463 with the amino acids KRPL (CmucAU1). EBOV GP harboring mutations in the endoproteolytic site, GP cl(-), has been previously described [20]. All constructs were cloned into the pCAGGS expression vector.

293T and HeLa cells were cultured in DMEM (Gibco) with 10% fetal bovine serum (HyClone) and penicillin/streptomycin (Gibco) at 37°C with 5% CO_2_. For flow cytometry and Western blotting, 293T cells were plated in 10 cm or 6-well plates one day prior to transfection. Cells were transiently transfected by Lipofectamine 2000 according to manufacturer's directions with 30 µg or 4 µg DNA per 10 cm plate or 6-well, respectively. Immunofluorescence microscopy was performed using HeLa cells that were plated on glass coverslips in 24-well plates and transfected with 1.5 µg DNA as above.

### Antigen-presenting and primary cells

Purified CD8 T cells from normal donors were obtained from the University of Pennsylvania Center for AIDS Research Immunology Core under a University of Pennsylvania IRB approved protocol. The human ovarian adenocarcinoma line OV79 has been described previously [48]. To create the OV79-SL9 antigen-presentig cells, OV79 cells were sequentially transduced to express HLA-A*02 [49] and a construct of GFP fused to a codon-optimized sequence of HIV-1 p17 Gag50–102. High titer lentiviral vectors were produced as described previously [50].

### Generation of SL9-specific CD8 T cells

Primary human CD8 T cells were cultured in X-Vivo 15 (Lonza) supplemented with 5% HABS (Valley Biomedical, Winchester, VA), 2 mM GlutaMax and 25 mM HEPES (Invitrogen). CD8 T-cells were transduced to express the SL9-specific HLA-A2 restricted 869TCR as described previously [51]. Transduction efficiencies were assessed by flow cytometric analysis of TRBV5-6 staining (anti-Vbeta5a, Thermo-Fisher) or HLA-A*02- SL9 tetramer stain (Beckman Coulter Immunomics).

### Stimulation and analysis of SL9-specific CD8 T cells

OV79-SL9 cells were plated at 16,000 cells/well on 48 well plates. After an overnight incubation cells were transduced with adenovirus expressing GFP (Ad GFP) or GFP and the EBOV Zaire glycoprotein (Ad GP) as described previously [12]. Briefly, adenoviruses were diluted in media and applied to cells at an MOI of 300. Media alone was used as a control. 48 h after transduction, target cells were analyzed for GFP and HLA expression. Floating and adherent cells, lifted by incubation with versene, were combined and stained for HLA-ABC or isotype control with APC-conjugated antibodies (BD-Biosciences). Alternatively, cells were stained for different MHC1 epitopes with W6/32 (eBiosciences), YTH862.2 (Santa Cruz Biotechnology), BB7.2 (BD Pharmingen), or GJ14 (Chemicon) primary antibodies, flowed by Alexa Fluor 647 (Invitrogen) secondary antibodies. 10,000 viable (forward scatter versus side scatter) events were collected on an LSR-II flow cytometer running BD FACSDiva-6 (BD-Biosciences), and analyzed in FlowJo (Tree Star Inc.).

SL9-specific TCR–transduced CD8 T cells were mixed with unmodified or adenovirally transduced OV79-SL9 target cells at a 2∶1 ratio for 1 h, followed by 4 h in the presence of brefeldin-A (Golgiplug, BD Biosciences). Stimulation with TPA (3 mg/ml, Sigma-Aldrich) and ionomycin (1 mg/ml; Calbiochem) with brefeldin-A was used as positive control. Cells were washed in PBS and surface-stained using CD8 conjugated to APC-H7, and then fixed and permeabilized with the Caltag Fix & Perm kit (Invitrogen) and stained using anti-TRBV5-6 FITC and macrophage inflammatory protein-1b (MIP-1b, CCL4)-PE. Sequential gates of 10,000 viable (forward scatter versus side scatter), CD8 positive events were acquired for all conditions on an LSR-II flow cytometer running BD FACSDiva-6 (BD-Biosciences). Data were analyzed for cytokine production in FlowJo (Tree Star Inc.).

### Cell lysates and Western blotting

Transfected cells were removed by resuspension in the culturing media. Cells were pelleted at 4°C for 3 min at 1300×g. Pellets were resuspended in 1% Triton X-100 or RIPA buffer with complete protease inhibitor cocktail (Roche) for 5 minutes. Lysates were cleared by centrifugation at 4°C at 20,800×g. 30 µl samples were mixed with reducing SDS buffer, boiled for 5 minutes, and separated on a 4–15% Criterion PAGE gel (Bio-Rad). Proteins were transferred to PVDF (Millipore) at a 400 mA constant current. Membranes were blocked in 5% milk in TBS. Membranes were probed with rabbit polyclonal anti-GP sera which recognizes the GP_1_ subunit [52], rabbit anti-AU1 antibodies (Bethyl labs), or anti-GAPDH monoclonal antibodies (Calbiochem) in blocking buffer. Protein was detected with stabilized goat anti- rabbit or mouse HRP conjugated antibodies (Pierce) in blocking buffer. Membranes were visualized with SuperSignal Femto substrate (Pierce).

### Flow cytometry

293T cells were detached from the plate 24 hours post transfection with PBS lacking Ca^++^ and Mg^++^ (−/−), 0.5 mM EDTA and combined with floating cells in culture media. Alternatively, floating cells in cluture media were removed and used exclusively (where indicated). Cells were pelleted at 4°C at 250×g, then resuspended in flow wash buffer (PBS −/− with 1% bovine calf serum and 0.05% NaAzide) and aliquoted for staining. For detection of EBOV GP, cells were stained with the human MAb, KZ52 [45] and detected with FITC anti-human IgG (PharMingen). For detection of AU1 epitopes, cells were stained with rabbit polyclonal anti-AU1 antibodies (Bethyl labs) and detected with FITC goat anti-rabbit IgG (Rockland). For detection of β1 integrin, cells were stained with anti-human CD29 PE-Cy5 conjugate (eBioscience); for detection of MHC1, cells were stained with anti- HLA-ABC PE-Cy5 conjugate (eBioscience). For intracellular staining, cells were permeabilized using Cytofix/Cytoperm (BD Biosciences) for 20 min on ice, followed by washing with Permwash (BD Biosciences). Antibodies where then diluted in Permwash buffer. For detection of GM130 and calnexin, mouse monoclonal FITC-conjugated antibodies were used (BD Transduction Labs). All staining was performed on ice, followed by washing. Live cell gates were drawn based on forward and side scatter. For each sample, 10,000 or 20,000 events in the live cell gate were collected and analyzed. Data were collected on a Becton Dickinson FACSCalibur and analyzed using FlowJo software (Tree Star, Inc.).

### Immunofluorescence microscopy

For HeLa cells, media was removed at 24 hours post-transfection, cells were washed with PBS and fixed with 3% PFA in PBS for 20 minutes. For non-adherent 293T cells, media containing floating cells was removed from plate, then centrifuged onto poly-D-lysine coated coverslips (BD Biosciences), then fixed. All samples were then washed with PBS, then permeabilized with 0.2% saponin, 1% goat serum in PBS for 5 minutes, then washed with PBS. Cells were blocked with 10% goat serum, 0.1% Tween-20 in PBS for 2 hours. For GP staining, coverslips were incubated with mouse anti-EBOV GP MAb 42/3.7 (gift from Yoshihiro Kawaoka) and detected with goat anti-rabbit Alexa Fluor 594 antibodies (Invitrogen). For AU1 staining, coverslips were incubated with rabbit anti-AU1 antibodies (Bethyl labs) and detected with anti-rabbit Alexa Fluor 488 antibodies (Invitrogen). Cells were washed with PBS after each staining step. Coverslips were mounted on glass slides with mounting medium containing DAPI (Vectasheild). Z-section images were collected on a Leica DMRE fluorescence microscope using Open Lab software (Improvision). Thirty z-sections per image were collected at 0.2 µm intervals. Z-section data were deconvoluted using Velocity software (Improvision) to a 98% confidence level or 15 iterations. Images shown are single, deconvoluted, z-sections.

### DTT treatment

At 24 hours post-transfection, sodium azide was added to 0.1% and 2-deoxy glucose was added to 10 mM. Cells were incubated an additional 30 min. Cells were then harvested and resuspended in flow wash buffer supplemented with 0.1% azide and 10 mM 2-deoxy glucose. DTT was then added to 150 mM and cells were incubated at 37°C for 20 minutes. Cells were then pelleted at room temperature and the supernatant was removed and blotted for GP as described above. Cells were then washed twice in flow wash and stained for flow cytometry as described above.

### Glycosidase treatment

At 24 hours post-transfection, floating cells were harvested and resuspended in 100 µl flow wash buffer. 100 U of neuraminidase (NEB) and/or 1000 U of PNGaseF (NEB) was then added. Cells were then incubated at 37°C for 20 minutes. Cells were then washed twice and aliquoted for flow cytometry or Western blotting as described above. Alternatively, cells were incubated with 2 mM benzyl-α-GalNAc (Sigma) or DMSO at 31°C for 48 hours. Cells were then given fresh media with 2 mM benzyl-α-GalNAc or DMSO and cultured at 37°C for 1 hour. Cells were then transfected as described above. At 24 hours post-transfection, floating and adherent cells were harvested and resuspended in 100 µl flow wash buffer. 1000 U of PNGaseF (NEB) or 2.5 mU of O-glycosidase (Sigma) was then added. Cells were then incubated and analyzed as above. For PNGaseF treatment of cell lysates, 30 µl of lysate was incubated with glycoprotein denaturing buffer (NEB) for 10 minutes at 60°C. Samples were then incubated with G7 buffer, NP40, and 500 U PNGase F (NEB) for 2 hours at 37°C, then blotted for GP as described above.

## Achnowledgments

The authors would like to thank Christian Fuchs for technical assistance, Erica Ollmann Saphire and Dennis Burton for providing the KZ52 antibody, Yoshihiro Kawaoka for providing anti-GP monoclonal antibodies, and Andrew Rennekamp and Rachel Kaletsky for helpful discussion.

## Supporting Information

Figure S1 EBOV GP masks multiple epitopes on MHC1. OV79 SL9 target cells were mock transduced or transduced with Adenoviral vectors expressing GFP (Ad GFP) or GFP and EBOV GP (Ad GP) at an MOI of 300. 48 h after transduction, cells were indirectly stained for different epitopes on MHC1 with primary antibody clones W6/32, YTH862.2, BB7.2 and GJ14 and detected with Alexa Fluor 647-conjugated secondary antibodies; isotype antibody =  grey peak; mock transduction =  blue trace; Ad GFP =  green trace; Ad GP =  orange trace. The approximate location of each epitope is marked by the yellow star in a cartoon depiction of MHC1 to the right of each graph. The W6/32 clone recognizes the MHC1 heavy chain and the β2 microglobulin. The YTH862.2 clone recognizes the α1 domain of the MHC1 heavy chain. The BB7.2 clone is specific for HLA-A2 and recognizes the α2 domain of the heavy chain. The GJ14 clone recognizes the β2 microglobulin.
(0.70 MB EPS)
